
JOURNAL INFORMATION
==============================
NLM Title Abbreviation: ScientificWorldJournal
Journal ID: ScientificWorldJournal
NLM Journal ID: 101131163
Journal ID: TSWJ

The Scientific World Journal

ISSN: 2356-6140
EISSN: 1537-744X
Publisher: Wiley

ARTICLE INFORMATION
==============================
PMCID: PMC6084274
PMID: 30147540
DOI: 10.1100/tsw.2001.251
Article ID: 975847
Article version: 1
Subjects: Short Report

Telomerase Allows Human Preosteoblasts to Maintain Their Phenotype during the Immortalization Process

Darimont Christian
Avanti Ornella
Tromvoukis Yvonne
Leone Patricia
Offord Elizabeth
Roberts Matthew A.
Pfeifer Andrea
Macé Katherine *
Nestle Research Center, P.O. Box 44, 1000 Lausanne 26, Switzerland
*Katherine Macé: catherine.mace@rdls.nestle.com
Publication date: 2001
Electronic publication date: 2001 Nov 7
Volume: 1
First page: 68
Last page: 68
Copyright: Copyright © 2001 Christian Darimont et al.
Copyright year: 2001
License: This is an open access article distributed under the Creative Commons Attribution License, which permits unrestricted use, distribution, and reproduction in any medium, provided the original work is properly cited.
License URL: https://creativecommons.org/licenses/by/3.0/

==============================
Miami *Nature Biotechnology* Short Reports
*TheScientificWorld* (2001) 1 (S3), 68SR
ISSN 1532-2246; DOI 10.1100/tsw.2001.251

### TELOMERASE ALLOWS HUMAN PREOSTEOBLASTS TO MAINTAIN THEIR PHENOTYPE DURING THE IMMORTALIZATION PROCESS

Christian Darimont, Ornella Avanti, Yvonne Tromvoukis, Patricia Leone, Elizabeth Offord, Matthew A. Roberts, Andrea Pfeifer, and Katherine Macé*
Nestle Research Center, P.O. Box 44, 1000 Lausanne 26, Switzerland.
* catherine.mace@rdls.nestle.com

**INTRODUCTION.** Normal human cells cultured *in vitro* have a limited proliferative lifespan, dividing a given number of times before they undergo a permanent growth arrest, known as replicative senescence. Transformation with oncogenes, such as Simian Virus 40 large T antigen (SV40 T-Ag), which bind and inactivate the protein products of the p53 and the retinoblastoma (pRb) genes, allow primary cells to extend their lifespan before undergoing a new growth arrest, known as crisis (1). Only rare cells survive the crisis and proliferate indefinitely as immortal cell lines. Due to increased chromosomal instability, these immortal cells lose part of their initial phenotype. Telomere maintenance, known to participate in chromosomal integrity, regulates the timing of cellular senescence (2). Combined with the inhibition of p53/pRb, telomere maintenance, via ectopic expression of the telomerase reverse transcriptase gene (TERT), allows human cells to escape from crisis and to become immortal (3). It nevertheless remains to be shown that this method of immortalization avoids the phenotypic drift of the cells. For this purpose, human preosteoblasts were first infected with SV40 T-Ag and subsequently with hTERT recombinant virus and phenotypic features of the cell lines were compared.

**RESULTS.** SV40 T-Ag expressing human periosteal (hPOB) cells divided about 80 times before undergoing a decline and then complete cessation of cell division at about p15. Cells expressing both SV40 T-Ag and hTERT (hPOB-tert cells) did not show any apparent crisis and are still growing after 62 passages, corresponding to about 575 divisions. Population doubling was 1.5 fold higher in hPOB-tert cells (15.2 hrs) as compared to hPOB cells (23.5 hrs). Semi-global evaluation of the influence of telomerase activity on the expression of genes involved in cell cycle or coding for senescence markers was performed using an in-house developed cDNA array comprising 483 probes selected to characterize this response. Analysis and validation are currently in progress, but initial results will be reported.

Karyotyping analysis indicate that the pre-immortalized hPOB cells (p10) and the immortalized hPOB-tert cells (p39) differ in their modal chromosome number, with a shift of near diploid metaphase toward hypotetraploidy. Increased chromosomal instability in hPOB-tert cells was not associated with an induction of c-myc oncogene expression. hPOB and hPOB-tert cells show similar capacity to differentiate into osteoblasts as determined by measurement of alkaline phosphatase activity, osteocalcin gene expression and mineralized nodule formation. Like the pre-immortalized cells, hPOB-tert cells were able to respond to bone anabolic agents such as dexamethasone and vitamin $D_3$.

**DISCUSSION.** This study demonstrates that telomerase activity allows pre-immortalized human preosteoblasts to escape from crisis and then to proliferate indefinitely as immortal cells. Interestingly, hTERT-induced immortalized preosteoblast cells maintain their ability to differentiate into osteoblasts although they display higher genomic instability and proliferative capacity than pre-immortalized cells. These results demonstrate the potential for using telomerase activity for the development of functional immortalized cells.

**REFERENCES.**

1. Smith, J.R. and Pereira-Smith, O.M. (1996) Science 273, 63-67
2. Bodnar, A.G., Ouellette, M., Frolkis, M., Holt, S.E., Chiu, C.P., Morin, G.B., Harley, C.B., Shay, J.W., Lichtsteiner, S., and Wright, W.E. (1998) Science 279, 349-352
3. Halvorsen, T.L., Leibowitz, G., and Levine, F. (1999) Mol. Cell. Biol. 19, 1864-1870
